# Supplementary material for: Large-scale prospective evaluation of co-folding across hundreds of Mac1–ligand complexes and three virtual screens
Source: eLife. 2026 Oct 12;15:RP110475. doi: 10.7554/eLife.110475 (PMC13657626; doi:10.7554/eLife.110475)
Supplement: Peer review history

# Editor report: eLife Assessment

**DOI:** 10.7554/eLife.110475.3.sa0

**Authors:** Fabrizio Marinelli

**Affiliations:** Medical College of Wisconsin United States

**Keywords:** Compelling, Important

This study addresses an **important** gap in drug discovery by delivering a rigorous, large-scale evaluation of widely used co-folding methods for predicting ligand-bound protein complexes and virtual screening. A key strength is the comprehensive benchmarking framework, which leverages structures and chemical compounds that were absent from the AI models training set, thereby providing particularly **compelling** and unbiased evidence of co-folding performance. The findings clearly delineate the complementary roles of deep learning-based co-folding and physics-based docking, offering practical guidance for their rational integration into drug discovery workflows. Overall, the conclusions are well supported by thorough analyses across a representative set of cases.

# Referee report: Reviewer #1 (Public review)

**DOI:** 10.7554/eLife.110475.3.sa1

The authors conducted a comprehensive benchmarking and evaluation of co-folding platforms, including AlphaFold3, Boltz-2, Chai-1, and the docking algorithm Dock3.7, which employs a physics-based scoring function that incorporates van der Waals interactions, electrostatics, and ligand desolvation energies. The system of interest was the SARS-CoV-2 NSP3 macrodomain (Mac1), an increasingly popular antiviral target, and the ligand sets comprised 557 unseen ligand poses (keeping the training for these co-folding platforms in mind). Additionally, the authors investigated whether the co-folding models could distinguish true ligands from non-binding small molecules. The study is thorough, with extensive statistical support and consensus across multiple metrics (chemoinformatics for quantifying ligand similarity and efficacy). The questions that the authors aim to address are whether the co-folding models struggle with memorization, whether they can distinguish between a true and a false binder, whether they replicate experimental binding affinities and efficacy, and how they compare to the physics-based docking algorithm (Dock3.7).

Strengths:

Overall, this is a scientifically solid paper.

The work is highly detailed and well executed, featuring thorough data analysis and statistical assessment.

Comments on revised version:

The authors have adequately addressed my concerns.

# Referee report: Reviewer #3 (Public review)

**DOI:** 10.7554/eLife.110475.3.sa2

Summary:

Core conclusions are well-supported by data: co-folding outperforms docking in known ligand pose/affinity prediction (validated by RMSD and IC₅₀ correlation), struggles with false positive discrimination in virtual screens (lower AUC values), and is complementary to docking (non-correlated errors, distinct strengths in drug discovery stages).

Strengths:

Unprecedented prospective design with 557 novel Mac1-ligand complexes ensures rigorous, independent evaluation of co-folding methods, provides an unbiased and rigorous benchmark dataset, which contains structures and compounds absent from the co-folding models training sets. Comprehensive comparison of 3 co-folding tools (AlphaFold3, Chai-1, Boltz-2) with DOCK3.7 across diverse targets and metrics enables nuanced performance assessment. The revised results clarify an intriguing finding: co-folding can predict correct ligand poses even when protein formations are mispredicted. The study clearly demonstrates complementary roles of co-folding (superior pose/affinity prediction for known ligands) and docking (better hit prioritization), and addresses deep learning memorization concerns via ligand similarity analysis.

Weaknesses:

The study identifies a major limitation of co-folding-failure to capture rare protein conformational changes, which deserve future investigation. The authors include uncalibrated Boltz-2 affinity data (addressing a prior comment) but note that large-scale free energy perturbation (FEP) comparisons are beyond their capabilities.

Appraisal of Aims Achieved:

The authors successfully achieved their primary aims and the results provide strong, well-supported evidence for their core conclusions. Key conclusions are grounded in the study's unbiased, training-set independent data, ensures the conclusions are not confounded by model memorization and are broadly applicable to the field's use of these co-folding models.

Field Impact:

This study provides a critical reality check for the field: co-folding models are powerful tools for pose prediction but are not yet standalone solutions for virtual screening, a key distinction that will prevent over-reliance on these models and guide more rational tool selection.

# Author comment: Author response

**DOI:** 10.7554/eLife.110475.3.sa3

**Authors:** Jongbin Kim, Galen J Correy, Brendan W Hall, Moira M Rachman, Olivier Mailhot, Takaya Togo, Ryan L Gonciarz, Priyadarshini Jaishankar, R Jeffrey Neitz, Eric R Hantz, Yagmur U Doruk, Maisie GV Stevens, Morgan E Diolaiti, Rashad Reid, Saumya Gopalkrishnan, Nevan J Krogan, Adam R Renslo, Alan Ashworth, Brian K Shoichet, James S Fraser

**Affiliations:** Department of Pharmaceutical Chemistry, University of California San Francisco, San Francisco, CA 94158 San Francisco United States; Department of Bioengineering and Therapeutic Sciences, University of California San Francisco, San Francisco, CA 94158 San Francisco United States; Department of Pharmaceutical Chemistry, University of California San Francisco, San Francisco, CA 94158 San Francisco United States; Department of Pharmaceutical Chemistry, University of California San Francisco, San Francisco, CA 94158 San Francisco United States; Department of Pharmaceutical Chemistry, University of California San Francisco, San Francisco, CA 94158 San Francisco United States; Department of Pharmaceutical Chemistry, University of California San Francisco, San Francisco, CA 94158 San Francisco United States; Department of Pharmaceutical Chemistry, University of California San Francisco, San Francisco, CA 94158 San Francisco United States; University of California, San Francisco San Francisco United States; Department of Pharmaceutical Chemistry, University of California San Francisco, San Francisco, CA 94158 San Francisco United States; Department of Pharmaceutical Chemistry, University of California San Francisco, San Francisco, CA 94158 San Francisco United States; Helen Diller Family Comprehensive Cancer Center, University of California San Francisco, San Francisco, CA 94158 San Francisco United States; Helen Diller Family Comprehensive Cancer Center, University of California San Francisco, San Francisco, CA 94158 San Francisco United States; UCSF San Francisco United States; Department of Biological and Medical Informatics, University of California San Francisco, San Francisco, CA 94158 San Francisco United States; Quantitative Biosciences Institute (QBI), University of California San Francisco, San Francisco, CA, 94158 San Francisco United States; University of California, San Francisco San Francisco United States; University of California, San Francisco San Francisco United States; University of California San Francisco San Francisco United States; UCSF San Francisco United States; Department of Bioengineering and Therapeutic Sciences, University of California San Francisco, San Francisco, CA 94158 San Francisco United States

The following is the authors’ response to the original reviews.

**Public Reviews:**

**Reviewer #1 (Public review):**

The authors conducted a comprehensive benchmarking and evaluation of co-folding platforms, including AlphaFold3, Boltz-2, Chai-1, and the docking algorithm Dock3.7, which employs a physics-based scoring function that incorporates van der Waals interactions, electrostatics, and ligand desolvation energies. The system of interest was the SARS-CoV-2 NSP3 macrodomain (Mac1), an increasingly popular antiviral target, and the ligand sets comprised 557 unseen ligand poses (keeping the training for these co-folding platforms in mind). Additionally, the authors investigated whether the co-folding models could distinguish true ligands from non-binding small molecules. The study is thorough, with extensive statistical support and consensus across multiple metrics (chemoinformatics for quantifying ligand similarity and efficacy). The questions that the authors aim to address are whether the co-folding models struggle with memorization, whether they can distinguish between a true and a false binder, whether they replicate experimental binding affinities and efficacy, and how they compare to the physics-based docking algorithm (Dock3.7).

We thank Reviewer 1 for this thoughtful summary of our work.

Strengths:

Overall, this is a scientifically solid paper. The work is highly detailed and well executed, featuring thorough data analysis and statistical assessment.

Weaknesses:

My main concern is that the study's aim is a bit unclear. Modern benchmarking studies comparing physics-based docking with deep learning-based co-folding approaches (e.g., AF3, Boltz-2, Chai-1, and others) are increasingly expected to go beyond aggregate performance metrics.

Indeed, we have gone into several examples of failures and successes for each of these methods. As we are not developing these methods ourselves, we also think this dataset will be a valuable contribution for improving them further.

In addition to rigorous dataset construction, transparent methodology, and appropriate statistical evaluation, high-impact benchmarks typically provide actionable guidance on when each method class is most appropriate, reflecting their distinct inductive biases and practical constraints. Failure-mode analyses that link performance differences to protein flexibility, ligand chemistry, or binding-site characteristics are particularly valuable, as they move comparisons beyond "scoreboard" assessments toward mechanistic understanding.

Right now, we do not observe meaningful trends that separate the failure modes for any individual method. This is covered in Supplementary Figures 6 and 7.

While full biological validation is not expected, qualitative interpretation grounded in physical and biological principles strengthens conclusions. Providing reproducible workflows or reference pipelines is not mandatory, but it is increasingly viewed as a best practice because it facilitates adoption and helps contextualize results for practitioners.

We note that our code is available (https://github.com/jongbin99/Cofolding/) and all structural data will be publicly accessible in the PDB alongside publication (we only held it back only for “blinding” during peer review to avoid contamination with any new deep learning methods).

**Reviewer #2 (Public review):**

Summary:

The manuscript by Kim et al. evaluates the performance of three modern AI-based methods in predicting complex structures and binding affinities between proteins and chemical compounds. An honest 'prospective' evaluation is achieved by studying benchmark structures and chemical compounds that did not exist in the PDB at the time the AI structure prediction models (AlphaFold3, Chai-1, Boltz-2) were trained.

Strengths:

(1) The study addresses an important question in modern computational biology and drug discovery, and establishes the strengths and limitations of the three tools in solving various computational chemistry tasks, including compound pose prediction, active-inactive discrimination, and potency ranking.

(2) The conclusions are based on examination of four separate targets and respective compound datasets, where for one of the targets, the authors also obtained numerous X-ray structures to serve as experimental answers for the binding pose prediction task.

(3) The study reports relationships between structure prediction confidence, predicted energies (DOCK3.7), and affinity predictions (Boltz-2) with the geometric accuracy of compound pose prediction as well as the experimentally measured potency.

(4) One of the key findings is the limited ability of co-folding methods to predict conformational rearrangements, which does not correlate with their ability to predict binding poses of the compounds inducing these rearrangements.

(5) The findings could serve as useful guidelines for computational chemists in selecting appropriate software and scoring schemes for each task.

We appreciate Reviewer 2’s summary of the novelty of the dataset and analysis.

Weaknesses:

While I consider this a solid study, several aspects would need to be addressed to make it really strong:

(1) DOCK3.7 docking and scoring experiments were performed using one experimental structure of Mac1, selected from dozens of structures based on a criterion that is not sufficiently well justified. For sigma2 receptor, dopamine D4 receptor, and AmpC β-lactamase, it is not clear which structures or models were selected for docking at all. It is well known that geometry predictions, scoring, and active-inactive ROC AUCs are all strongly influenced by the selected structure. It would be important to attempt Mac1 docking using all available experimental Mac1 structures, or at least against representative structures in various conformations; it would also be quite insightful to compare results to docking of the same compound sets to AF3, Boltz-2 and Chai-1 predicted structures of Mac1. Same goes for the docking studies of sigma2, D4, and AmpC β-lactamase.

In any program, a decision has to be made as to which template will be used for docking, we justified the choice in the methods:

“We used this structure because the inhibitor (Z5014193706) was the most potent molecule with a structure determined around the same time as the ligands in this dataset were tested.”

We stand by this as a reasonable assumption. Similarly, for sigma2, D4, and AmpC β-lactamase, the template was chosen in the respective papers:

a) The σ_2_ receptor bound to cholesterol (PDB ID: 7MFI) was used in the docking calculations.

- This structure was determined in the paper, the first structure of sigma2 and therefore a worthy template

b) The D4 receptor campaign used PDB 5WIU

- This was one of two D4 structures available and chosen because it was not bound to sodium

c) For AmpC, the campaign used the structure in the Protein Data Bank (PDB) 1L2S

- This maximizes comparisons to other docking studies that used the same receptor template.

The major goal of this study is to compare different methods under reasonable (but perhaps as the reviewer points out, not optimal) conditions, not to optimize docking score.

(2) For binding affinity predictions, as a control, authors should consider compound co-folding with an unrelated protein, or even with a pseudo-peptide that consists of a few random single amino acids - this would provide an honest baseline for such predictions.

This suggestion would be valuable for understanding the performance for these methods from the perspective of ligand specificity (a valuable, but separate, goal). Surely this will generate some number or some prediction - but what would this baseline mean and how would it be relevant for drug discovery? Therefore, we do not think this suggestion is relevant for the issues being investigated in this manuscript.

(3) ROC curves Figure 3 and elsewhere should be shown, and AUCs quantified/reported on a log or square-root scaled x-axis, to emphasize early enrichment, which is the area of practical significance for these predictions. For example, Figure 3A currently suggests that the pose prediction performance of AF3 exceeds that of Boltz-2 whereas the early enrichment is clearly better for Boltz-2.

We agree with this, and added a semi-logAUC plot for Figure 3A. For Figure 5, we also generated a semi-logAUC plot to see early ligand enrichment clearly, added as Supplementary Figure 11. We added the text:

“Considering its early enrichment performance, Boltz-2 Ligand ipTM was the strongest predictor of pose accuracy based on normalized logAUC (20.5% above random, Fig. 3a). In contrast, although Boltz-2 pIC_50_ showed poor overall discrimination, it overestimated its ability to enrich true positive poses at low false positive rates, despite having a weak early enrichment behavior”

(4) 'Trained set' in figures and text should probably be 'training set'? Or otherwise explain this new term the first time it is introduced.

Thank you for pointing out this for clarification. ‘Training set’ is the correct word, and we made changes appropriately across all figures and texts.

(5) Figure 1 illustrates a projection onto the first two principal components of a space that apparently had only one (scalar) metric for each compound pair (% maximum common substructure or Tanimoto coefficient); the authors need to better explain the principle behind this analysis and visualization.

This suggestion is valuable, since we often use PCA to reduce dimensionality for more complex features. For clarification, we actually have a full pairwise similarity matrix for all tested Mac1 compounds based on each of Tc and MCS%. PCA for each MCS% and Tc is a representation of each pairwise similarity matrix. We also made a change in Figure 1 caption to make this point clearer:

“projection of compounds represented by their full pairwise similarity vectors (by ECFP-4 Tc and MCS%)”

**Reviewer #3 (Public review):**

Summary:

This study's core conclusions are well-supported by data. It is shown that co-folding outperforms docking in known ligand pose/affinity prediction (validated by RMSD and IC₅₀ correlation), struggles with false-positive discrimination in virtual screens (lower AUC values), and is complementary to docking (non-correlated errors, distinct strengths in drug discovery stages).

Strengths:

(1) Unprecedented prospective design with 557 novel Mac1-ligand complexes ensures rigorous, independent evaluation of co-folding methods.

(2) Comprehensive comparison of 3 co-folding tools (AlphaFold3, Chai-1, Boltz-2) with DOCK3.7 across diverse targets and metrics enables nuanced performance assessment.

(3) The study clearly demonstrates complementary roles of co-folding (superior pose/affinity prediction for known ligands) and docking (better hit prioritization), and addresses deep learning memorization concerns via ligand similarity analysis.

We thank Reviewer 3 for pointing out the unprecedented and comprehensive nature of our study

Weaknesses:

(1) Limited generalization to diverse protein families (e.g., no ion channels/transporters).

We agree - we have not explored the entire proteome and these are important target classes that will surely be investigated by future studies. We focused on targets here where we had large number of X-ray crystal structures (Mac1) and affinity/inhibition measurements from docking (the other three targets).

(2) Ambiguity in the mechanism underlying co-folding's failure to predict rare conformational changes.

Again, we agree. We are not the developers of these methods. We observe that these methods do not predict conformational changes with high fidelity and this weakness is an area that co-folding methods will surely prioritize in the future.

(3) Virtual screen comparison is unbalanced (docking-prioritized hit lists bias results).

We acknowledge this in the results: “An important caveat is that the hit-lists were composed of molecules prioritized by docking in the first place, giving it an advantage on these particular sets.” and discussion: “Finally, comparing co-folding to docking based on hit-lists themselves selected by docking is arguably unfair to co-folding. Counter-balancing this is the inclusion, in each of the three hit lists, of molecules that had mediocre and poor docking scores intentionally selected to test the correlation between docking score and hit-rate. Here too, the correlation between co-folding score and likelihood to bind, what we sometimes call a “dock-response-curve” was no better than docking’s, often worse (SFig.11).”

**Recommendations for the authors:**

**Reviewer #1 (Recommendations for the authors):**

Here are suggestions for revisions:

(1) The writing is at times obtuse and hard to follow.

This happens sometimes when multiple authors are writing together. We apologize and are happy to respond to specific areas that can be streamlined to be easier to follow.

(2) In the Results section, "A set of 557 previously unreported Mac1 ligand complexes", the authors have compared the ligand poses across different metrics such as Tc - a standard, highly effective method in chemo-informatics and MCS (maximum common substructures); these are standard metrics for quantifying the structural similarity between pairs of small molecules. This part of the analysis checks whether this is memorization; it is critical to compare the two metrics, but it is not sufficient to draw a conclusion.

Thank you for pointing out about the structural similarity of molecules co-folded to those present in the training set (resolved as Mac1 complexes and deposited in PDB before training dates). We have conducted an analysis where we do a pairwise similarity comparison for all ligands present in the PDB (regardless of the target), by both Tc and MCS, and overlay the cluster of ligands we tested (Mac1, AmpC, sigma2, D4). This should show where our tested benchmark datasets lie in the chemical space covered in the entire PDB. Each cluster (around 500 to 1300 compounds per target system) is overlaid on the cluster of all ligands deposited in PDB (over 50,000 compounds), and each cluster was relatively diverse by both Tc and MCS.

(3) In the "Co folding can accurately reproduce poses of ligands dissimilar to those trained." Subsection under Results, the authors' conclusions are hard to follow; they state that the co-folding models often mispredict or miss the alternative conformation, but they also predict poses that are distinct from the training set. What does that imply?

Our interpretation is actually a somewhat unsettling one: co-folding gets the ligand pose right even when it gets the protein wrong, and even when the ligand is novel. This suggests the models may be anchoring on conserved pharmacophoric interactions (like the adenosine-mimicking purine scaffold) rather than truly modeling the physics of the full complex. We added to the results section:

This result suggests that co-folding reliably recapitulates dominant ligand-binding interactions even in the absence of accurate protein conformational modeling, providing further support to the idea that they are learning specific interaction patterns rather than a deeper physics-based representation (Masters et al. 2025).

(4) The Discussion section connects the results and conclusions, but it can be challenging to grasp the study's overall message.

We think the final paragraph hits on three major points:

- Co-folding accurately predicts ligand poses for known binders, but fails to capture conformational changes

- Co-folding does not reliably distinguish true binders from false positives in virtual screening hit lists

- Docking and co-folding are complementary rather than competing tools

(5) The work is highly detailed and well executed, featuring thorough data analysis and statistical assessment. The value of the paper would be further enhanced by explaining how it differs from seemingly similar results reported in other studies, including the one cited in this manuscript (see https://www.biorxiv.org/content/10.64898/2025.12.04.692352v1).

The Mac1 results are completely unique. However, the docking datasets are exactly the same as those analyzed in the Menon et al manuscript. We don’t think our results differs from conclusions of the Menon et al manuscript as we wrote: These observations are supported by a fascinating study on some of the same ligand sets as investigated here, using AlphaFold3, reaching similar conclusions (Menon et al. 2025).

**Reviewer #3 (Recommendations for the authors):**

(1) Expand target diversity to include ion channels, transporters, etc., beyond enzymes and GPCRs.

(2) Investigate the cause of co-folding's failure in predicting rare conformational changes (e.g., adjust sampling, MSA inputs, or add experimental constraints).

(3) Mitigate docking bias in virtual screens (e.g., re-analyze unbiased compound libraries).

We addressed these three points in the public review above

(4) Test Boltz-2's affinity predictions without linear calibration and compare with FEP.

The data without linear calibration are included in the manuscript. Comparing such a large number of compounds with FEP is currently beyond our capabilities.

(5) Conduct proof-of-concept to test co-folding-docking integration for better hit rates.

We think this is well beyond the scope of this manuscript - but look forward to testing this idea in the future.

We also got one community review that we respond to below:

Summary

This manuscript evaluates the performance of co-folding models when tasked with (1) the recapitulation of a large number of experimentally determined co-crystal structures of Mac1 with a series of Mac1 ligands and (2) the rescoring of hits to identify false positives originally derived from a set of large docking-based virtual screens. The evaluation leverages a dataset of crystal structures and affinity data from high-throughput crystallographic and biophysical screens, respectively. These data uniquely enable this report to focus on the ability of co-folding models to handle ligands, resulting in an analysis that is particularly timely given the wide adoption of co-folding models and the relative scarcity of such ligand-focused benchmarks among existing evaluations, which have primarily focused on protein structure prediction or binder design.

Thank you for this thoughtful summary of our work

Feedback

The experiments and analyses in the manuscript are well thought-out and do not have any significant issues. There are a few high-level points that may improve the clarity and completeness of the results. Importantly, none of the suggested additional experiments will affect the conclusions of the paper, but rather help provide additional context for the results:

The first section presents an exciting opportunity to frame the Mac1 ligands against ligands in the PDB more broadly. It would be informative to assess whether chemotypes that are easier or harder to predict accurately and confidently are over- or under-represented in the PDB as a whole. Note that this is not a recommendation that new scaffold similarity metrics be incorporated into the analysis, but rather that analyses similar to those already performed in the manuscript are performed using all ligands in the PDB. For example, PCA-based analyses similar to those in Fig. 1c could be used to examine Mac1 ligands in the context of all PDB ligands enabling questions such as whether similarity to a nearest PDB neighbor, cluster size in a Tc/MCS PCA space, or other frequency-based measures show any relationship with prediction vs. crystal structure RMSD. Such analyses could provide additional insight into how effectively models leverage ligand information present in the PDB overall, as opposed to biases arising specifically from scaffolds represented in Mac1 structures in the PDB, which are already well covered in the manuscript. The conclusion that Tc/MCS do not correlate with the ligand RMSDs for the ligands already associated with the Mac1 is well supported, and presumably suggests that a correlation would not exist against the backdrop of the PDB, but it would be interesting to see the data using analyses similar to those already done in the manuscript nonetheless.

We are adding new figures in SFig.1 that consider how different clusters of ligands tested for our co-folding analysis are distributed across the chemical space in PDB. This is done by making a similarity comparison between every ligand in PDB and those tested in our analysis by Tc and MCS%, then plotting in PCA space for each metric. We are excited to see that each dataset covers a wide scope in PCA space, but at the same time, there are unexplored areas in the chemical space of PDB by co-folding.

Similarly, even though the four proteins used in this manuscript are not themselves the primary focus of the analysis, it would be valuable to perform a high-level assessment of the precedent for each protein in the PDB (beyond the count of liganded structures in Table S6), either in protein sequence space (e.g., MSAs) or structural space (e.g., FoldSeek). An analysis like this would provide important context about whether any of the proteins in the study have close homologs with liganded structures in the PDB, or are generally overrepresented in the PDB. The fact that the AUC for L-pLDDT for AmpC is higher than σ_2_ and D4, for example, is notable given the relative abundance of liganded AmpC structures in the PDB (this raises potentially interesting questions related to where DOCK3.7 and AF3 actually place the ligands, given the orthosteric β-lactam binding pocket in AmpC, although this is outside of the scope of this manuscript).

High-level assessment of the precedent for each protein in the PDB will definitely help to understand if proteins we used have close homologs with liganded structures in the PDB. Our Supplementary Table 6 covers the extent to which these liganded structures were available by cutoff dates for AF3, Chai-1 and Boltz-2. AmpC had more homologs than sigma2 and D4, and this may explain a better AUC for AF3 L-pLDDT specifically for this target.

A discussion of the affinity probability results (`affinity_probability_binary`) from Boltz-2 is likely warranted in the second section in addition to the pIC_50_s that are already reported (`affinity_pred_value`). The former seems like it would be more applicable for section 2 of the manuscript, but both warrant inclusion—they should both be calculated by default when the affinity pipeline in Boltz-2 is turned on, so it wouldn't involve any more inference.

As boltz-2 affinity module outputs both affinity probability binary output and affinity predicted value, we kept track of both metrics. So we tried re-ranking hit lists using both metrics. Where boltz-2 performed better (Sigma2, D4), binary probability values were more representative as a metric to differentiate true actives from non-binders. This was more clear in semi-logarithmic ROC plots. However, in AmpC, both Boltz-2 scoring metrics performed similarly. Such inconsistency in trend made it difficult to draw conclusions.

Minor points

A more detailed description of the experimental methods used to generate the ground-truth data in the introduction (even though these have been explained in prior works) would help orient the reader early on, and ground the benchmarking aspect of the story. In general, the abstract and introduction would benefit from a more cohesive through-line to tie the two complementary but orthogonal sections of the paper together.

We will include a more thorough description alongside the PDB depositions. As for the two sections, we have tried to tie them together from the perspective of drug discovery workflows…

The cutoffs in the "Co-folding can accurately reproduce..." section shift between 2.5 Å (from the ligand center of mass) and 2.0 Å. Is there a reason for this? Along similar lines, mentioning cutoffs for true positives/negatives when introducing the ROC analyses later on in the Mac1 section seems unnecessary since no cutoff should be necessary here.

We used 2.5A distance to COM to just get at “broadly the correct binding site” for fast filtering and 2.0A RMSD because that is the broadly accepted standard in the field for “relatively correct binding pose”.
